# Molecular mechanisms underlying AMH elevation in hyperoestrogenic states in males

**DOI:** 10.1038/s41598-020-71675-7

**Published:** 2020-09-15

**Authors:** Clara Valeri, María M. Lovaisa, Chrystèle Racine, Nadia Y. Edelsztein, Marina Riggio, Sebastián Giulianelli, Marcela Venara, Patricia Bedecarrás, María G. Ballerini, Nathalie di Clemente, Caroline A. Lamb, Helena F. Schteingart, Rodolfo A. Rey

**Affiliations:** 1 Centro de Investigaciones Endocrinológicas “Dr. César Bergadá” (CEDIE), CONICET-FEI-División de Endocrinología, Hospital de Niños Ricardo Gutiérrez, C1425EFD Buenos Aires, Argentina; 2 Sorbonne Université, INSERM, Centre de Recherche Saint Antoine (CRSA), 75012 Paris, France; 3 Institut Hospitalo-Universitaire ICAN, 75013 Paris, France; 4 Sorbonne Paris Cité, Paris-Diderot Université, 75013 Paris, France; 5 Instituto de Biología y Medicina Experimental (IBYME-CONICET), C1428ADN Buenos Aires, Argentina; 6 Instituto de Biología de Organismos Marinos, IBIOMAR-CCT (CENPAT-CONICET), U9120ACD Puerto Madryn, Argentina; 7 Departamento de Biología Celular, Histología, Embriología y Genética, Facultad de Medicina, Universidad de Buenos Aires, C1121ABG Buenos Aires, Argentina

**Keywords:** Hormone receptors, Growth disorders

## Abstract

Anti-Müllerian hormone (AMH) is secreted by Sertoli cells of the testes from early fetal life until puberty, when it is downregulated by androgens. In conditions like complete androgen insensitivity syndrome (CAIS), AMH downregulation does not occur and AMH increases at puberty, due in part to follicle-stimulating hormone (FSH) effect. However, other conditions like Peutz-Jeghers syndrome (PJS), characterised by low FSH, also have increased AMH. Because both CAIS and PJS may present as hyperoestrogenic states, we tested the hypothesis that oestradiol (E2) upregulates AMH expression in peripubertal Sertoli cells and explored the molecular mechanisms potentially involved. The results showed that E2 is capable of inducing an upregulation of endogenous AMH and of the *AMH* promoter activity in the prepubertal Sertoli cell line SMAT1, signalling through ERα binding to a specific ERE sequence present on the h*AMH* promoter. A modest action was also mediated through the membrane oestrogen receptor GPER. Additionally, the existence of ERα expression in Sertoli cells in patients with CAIS was confirmed by immunohistochemistry. The evidence presented here provides biological plausibility to the hypothesis that testicular AMH production increases in clinical conditions in response to elevated oestrogen levels.

## Introduction

Anti-Müllerian hormone (AMH), also known as Müllerian inhibitory substance (MIS), is a homodimeric 140-kDa glycoprotein^1^, which belongs to the transforming growth factor β (TGFβ) superfamily. It is encoded by a gene of approximately 2.7 kbp^2,3^ located on human chromosome 19p13.3^4^. The most biologically relevant and irreplaceable function of AMH takes place during sex differentiation in early fetal life, when testicular AMH secreted by Sertoli cells^5^ induces the regression of the paramesonephric Müllerian ducts, which otherwise develop to give rise to the Fallopian tubes, the uterus and the upper portion of the vagina^6,7^. Although Müllerian duct regression is completed early in gestation, Sertoli cells continue to produce AMH until adulthood, and granulosa cells of the ovarian follicles also secrete AMH from the second half of gestation^8^ until menopause^9,10^.

In the fetal Sertoli cell, *AMH* gene expression is triggered by the nuclear transcription factor SOX9^11,12^ and subsequently upregulated by transcription factors SF1^11,13–15^, GATA4^14,16,17^ and WT1^18^. Thus, high levels of AMH are secreted during the period of sex differentiation independently of pituitary gonadotrophin action on Sertoli cells. Later, follicle-stimulating hormone (FSH) produced by the pituitary, provokes a surge in AMH testicular output^19–21^. The molecular pathway induced by the seven-transmembrane domain FSH receptor present on the Sertoli cell membrane involves the Gsα subunit^22^, cyclic AMP and protein kinase A, which finally activates the expression and/or nuclear translocation of transcription factors SOX9, SF1, AP2 and NFκB^23–25^. During pubertal development, testicular AMH production declines^26^ as a consequence of the increase of the intratesticular concentration of testosterone^19,27^ and the emergence of meiotic spermatocytes^19,28^. Androgens downregulate AMH expression at the transcriptional level by activating the androgen receptor (AR) which, in turn, blocks SF1-induced transactivation of the AMH promoter^29,30^.

During the fetal period and early postnatal life, the elevated levels of androgens existing in the testes are unable to downregulate AMH expression because Sertoli cells do not express the AR at that time^31–34^. Accordingly, AMH levels do not decrease at pubertal age in mice with disrupted androgen signalling^19,35^ and in humans with defective androgen synthesis^36–38^ or with a complete androgen insensitivity syndrome (CAIS) due to AR mutations^36–41^. In these patients, testes differentiate but virilisation fails; thus, they are born with female external genitalia and raised as girls. The diagnosis is made due to the absence of menses at puberty, and in the past, orchiectomy was usually performed^42^. Interestingly, at pubertal age testicular AMH increases^36,37^ concomitantly with FSH and oestradiol levels. FSH can undoubtedly be responsible for the increase in AMH. However, a temporal coincidence in the elevation of AMH and oestradiol (E2) also occurs in boys and adolescents with Peutz-Jeghers syndrome^43^, characterised by the existence of Sertoli cell proliferations overproducing oestrogens that lead to low FSH levels^44^, and thus cannot explain the increased AMH levels. Interestingly, the human *AMH* promoter contains a consensus sequence for a half-oestrogen response element (ERE)^45^, and oestradiol has been shown to modify AMH expression in the adult ovary^46^. Altogether, these observations raise the possibility that the hyperoestrogenism observed in CAIS and Peutz-Jeghers syndrome may account for the elevation in testicular AMH production.

Oestrogens are steroid hormones synthesised from androgens by aromatase, a member of the cytochrome P450 superfamily encoded by only one gene, *CYP19A1*, in humans^47^. During development, aromatase is expressed in the human neonatal and infant testis^31^, and in the rat testis, aromatisation of androgens to oestrogens is stimulated by the action of FSH on prepubertal Sertoli cells^48^. In humans with Klinefelter syndrome, elevated FSH is also presumed to increase aromatase activity in Sertoli cells^49^, thus resulting in increased oestrogen levels and gynaecomastia^50^. E2 signals by binding to classical intracellular oestrogen receptors or to a membrane oestrogen receptor, known as GPER, GPER1 or GPR30^51^. The classical oestrogen receptors α (ERα) and β (ERβ) belong to group A of the nuclear receptor subfamily 3, with binding capacity on DNA sequences known as EREs. The DNA-binding domains of ERα and ERβ have an identity of 97%, whereas the homology of the ligand-binding domains does not exceed 60%; both bind to E2 with a similar affinity^52,53^ but differences in this region result in variable affinity to diverse synthetic compounds^54^. GPER belongs to the family of seven-transmembrane domain, G protein-coupled receptors (GPCRs), which classically mediate rapid cellular responses involving kinases, ion channels, and second messengers^55^. GPER is predominantly localised in the membranes of the endoplasmic reticulum^56,57^.

Further than synthesising oestrogens, the testes express the different oestrogen receptors, with an ontogeny and expressiveness that vary according to the species studied^58^. All three ERs have been identified in the different human testicular compartments^31,59–61^. Although the information on developmental expression of the three ERs in the male gonad is scant, in rodent Sertoli cells ERβ seems to be predominant over ERα in the adult, whereas the opposite occurs in peripubertal ages^62^. GPER is present in Sertoli cells from the time of pubertal onset^63^. Clinical observations in humans and experimental models indicate that ERα is required for spermatogenesis and maintenance of sperm morphology and motility^64,65^ while ERβ is required to induce the fate of testicular cells in the undifferentiated gonad^66^. GPER mediates direct effects of oestrogens on germ cells in mice^67^. Finally, all ERs seem to be involved in proliferation and maturation of Sertoli cells in rodents and boar^62,63,68^. Little is known about the direct effects of oestrogens in the prepubertal human testis^69^. While no major clinically evident testicular dysfunction has been observed in males with genetic forms of aromatase deficiency^70^, a patient with oestrogen insensitivity due to an ERα defect had high serum E2 and gonadotropin levels, but low AMH and inhibin B, suggesting a potential deficiency in Sertoli cell function^71^.

In this work, we tested the hypothesis that E2 upregulates AMH expression in peripubertal Sertoli cells, thus explaining the increased levels of AMH observed in paediatric patients with hyperoestrogenic states, and explored the molecular mechanisms potentially involved.

## Results

### Presence of ERα and ERβ in testes of patients with androgen insensitivity

**Figure 1:**
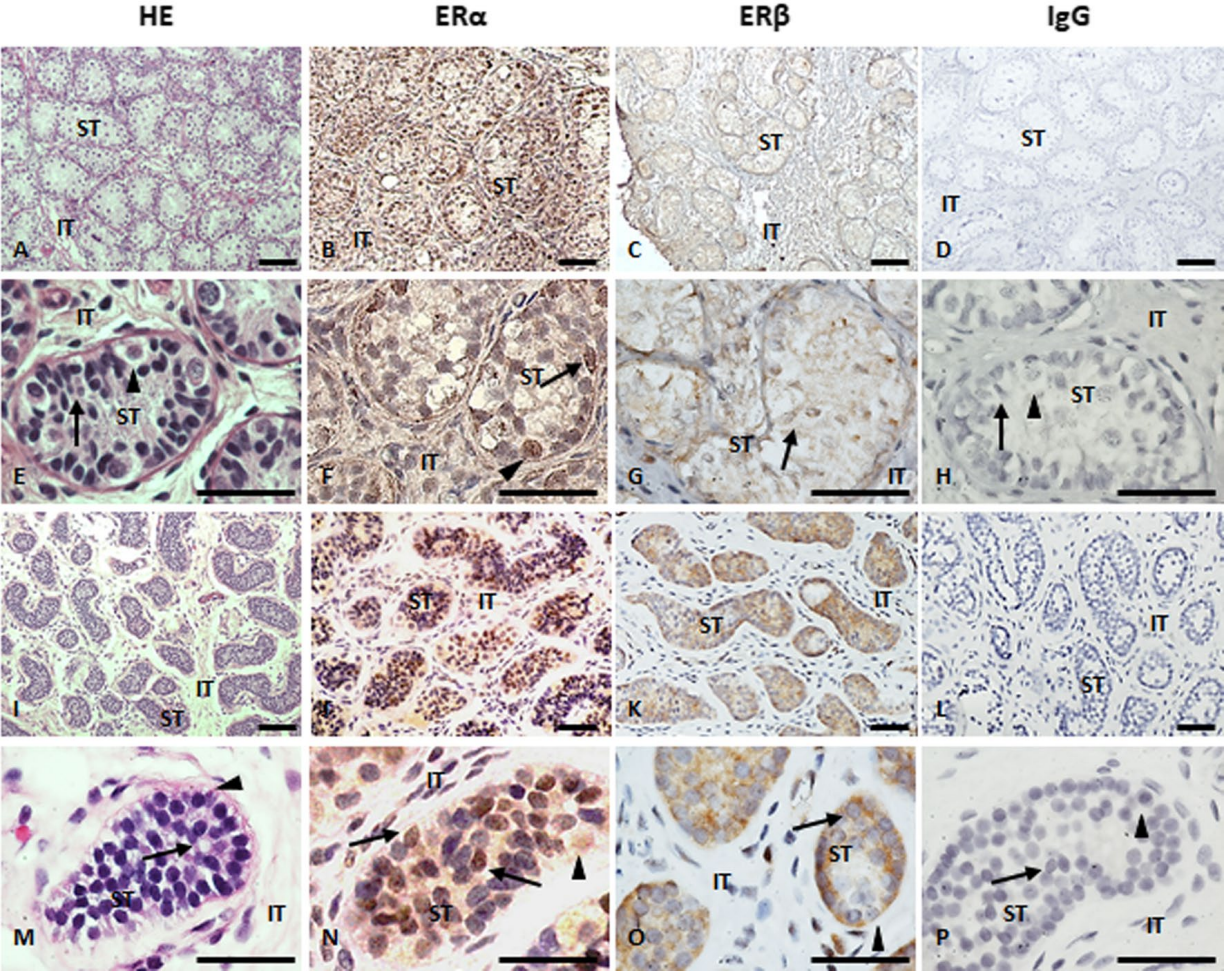
Immunohistochemistry for ERα and ERβ. A-H: Normal prepubertal human testis (biopsy showing unaffected tissue from a 7-year-old boy with acute lymphoblastic leukaemia). I-P: Testis from a 6-year-old patient with complete androgen insensitivity syndrome (CAIS). *HE* haematoxylin–eosin, *IgG* primary antibody was replaced by IgG from nonimmune serum (negative control), *IT* interstitial tissue, *ST* seminiferous tubule; arrows: Sertoli cells (ovoid or elongated nuclei); arrowheads: germ cells (spermatogonia, round nuclei and abundant pale cytoplasm). Detection was performed using HC-20 antibody for human ERα, and Ab1531 for human ERβ. The bars represent 60 µm.

To address the physiological and pathophysiological relevance of our hypothesis in humans, we first assessed the presence of ERα and ERβ in testicular tissue of 46,XY patients with CAIS, an hyperoestrogenic condition. Immunohistochemistry of testicular tissue obtained from archival samples of gonadectomy from four patients with CAIS showed the presence of ERα and ERβ in Sertoli cells (Fig. 1). We compared this expression pattern with that observed in four biopsies of prepubertal human testes without injury, obtained from archival samples of paediatric patients with acute lymphoblastic leukaemia in which absence of gonadal involvement was verified. We detected the presence of ERα and ERβ in the Sertoli cells of normal testicular tissue and of patients with CAIS, which had scarce germ cells in the seminiferous tubules (Fig. 1). Although immunohistochemistry is not quantitative, ERα staining seemed somewhat more intense than that of ERβ in cell nuclei. These results indicate that Sertoli cells of patients with CAIS express oestrogen receptors, setting the basis for a potential AMH increase in response to elevated oestrogen levels.

### Effect of anti-oestrogen treatment on serum AMH in prepubertal male mice

**Figure 2:**
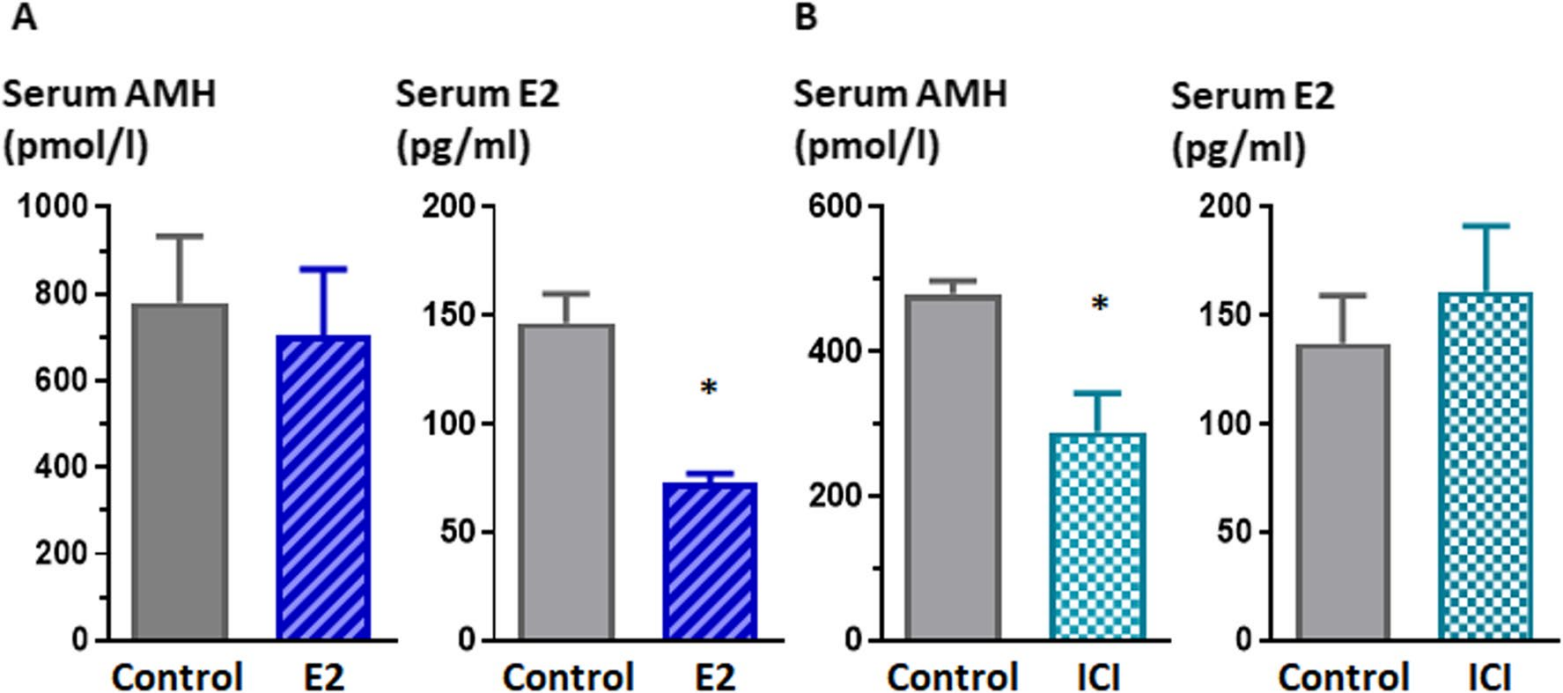
Serum AMH and E2 levels in mice. **(A)** Serum levels of AMH and E2 in 9-day-old male mice treated sc with E2 (20 µg/day) or vehicle from postnatal day 4 to day 8. **(B)** Serum levels of AMH and E2 in 9-day-old male mice treated sc with ICI 182780 0.8 mg (single dose) or vehicle on postnatal day 4. * *P* < 0.05, Student's t-test for unpaired samples, n = 8.

To test the effect of E2 on AMH production before pubertal testis maturation, we treated male CF-1 mice with E2 from postnatal day 4 to day 8, and measured serum AMH and E2 levels on postnatal day 9. We observed no significant changes in serum AMH as compared to control mice, but we noted that the treatment protocol could not achieve increased E2 levels but rather reduced them (Fig. 2A), so it was uninformative. As an alternative strategy, we treated 4-day-old male mice with a single dose of the ERα- and ERβ-antagonist ICI 182780. At 9 days of age, we observed a significant decrease in serum AMH as compared to control mice (Fig. 2B). These results suggest that blocking the ER pathway causes a decrease in testicular AMH output, in line with our hypothesis that E2 stimulates the secretion of AMH.

### AMH expression in response to various E2 doses in SMAT1 Sertoli cells expressing ERα or ERβ

**Figure 3:**
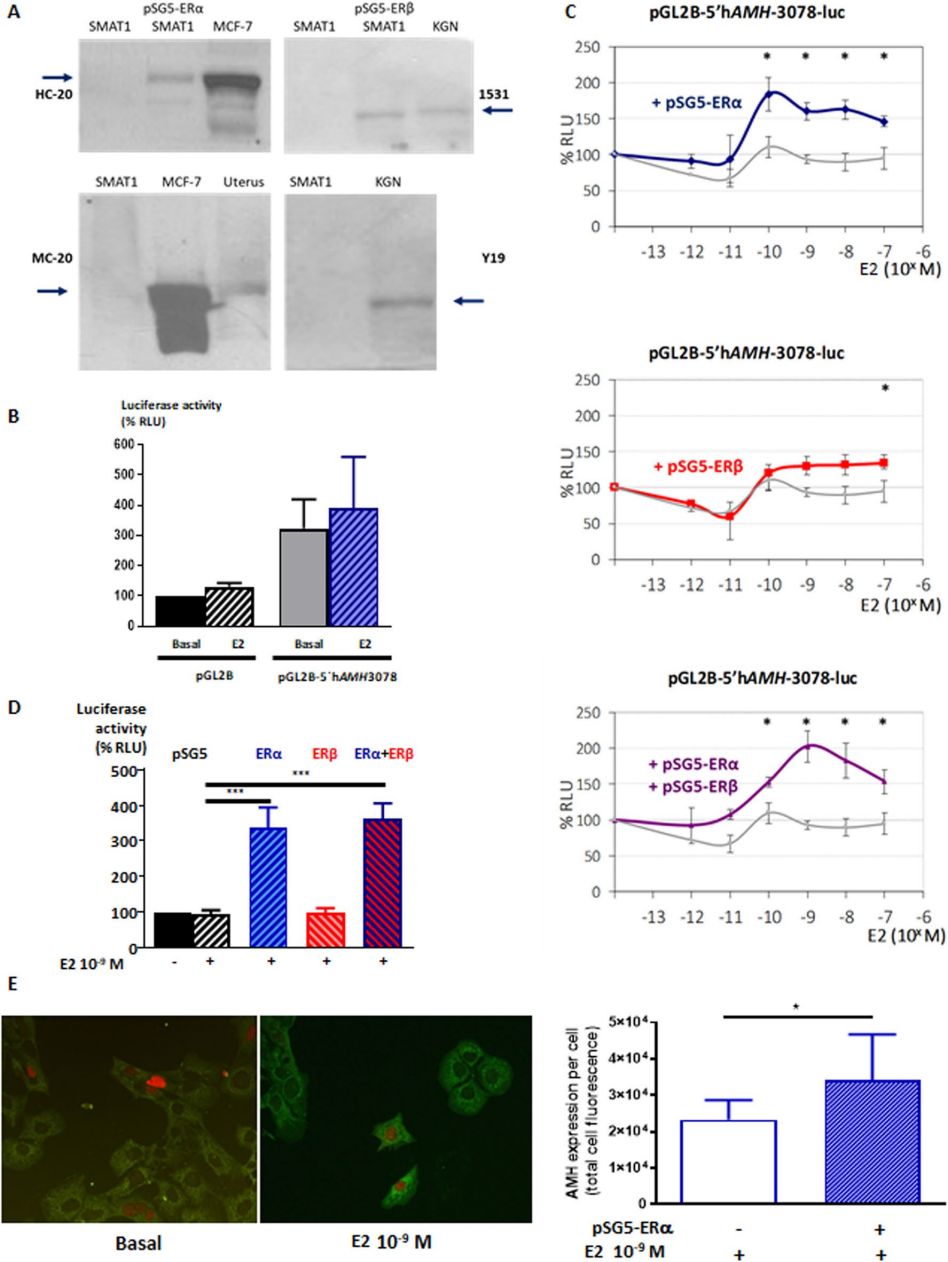
Luciferase assays to assess human *AMH* promoter (3,078 bp) activity in SMAT1 cells. **(A)** Western blot for ERα (left) and ERβ (right) in SMAT1 cells, non-transfected or transfected with pSG5-ERα or pSG5-ERβ, in MCF-7 cell line and uterus—that endogenously express ERα—and in KGN cell line endogenously expressing ERβ. Detection was performed using the following antibodies: HC-20 (human ERα), MC-20 (murine ERα), 1531 (human ERβ) and Y-19 (murine ERβ). Representative of 3 experiments. (**B)** Cells transfected with pGL2B or pGL2B-5′hAMH-3078, but not transfected with ER expression vectors were exposed to E2 10^–9^ M or basal medium. % RLU: relative luciferase units, considering the baseline condition (pGL2B without E2) as 100%. Student's t-test for paired data, n = 4. **(C)** Cells transfected with pGL2B-5′hAMH-3078, and co-transfected with pSG5-ERα, pSG5-ERβ or both, were exposed to E2 from 0 to 10^–7^ M. The grey curve repeated in all figures corresponds to SMAT1 cells not transfected with ER vectors. % RLU: relative luciferase units, considering the baseline condition (without ER and without E2) as 100%. * *P* < 0.05 ERα or ERβ or ERα + ERβ vs no ER, Student's t-test for paired data, n = 4. **(D)** Cells transfected with pGL2B-5′hAMH-3078, and co-transfected with pSG5, pSG5-ERα, pSG5-ERβ or both, were exposed to E2 10^–9^ M or basal medium. % RLU: relative luciferase units, considering the baseline condition (without ER and without E2) as 100%. *** *P* < 0.001 ERα or ERβ or ERα + ERβ vs no ER, analysis of variance (ANOVA), followed by Tukey's multiple comparison test, n = 8. **(E)** Endogenous expression of AMH protein in SMAT1 cells transfected with ERα and exposed to E2 10^-9^ M. The intensity of AMH immunofluorescence (green) was compared between SMAT1 cells effectively transfected with pSG5-ERα (red) and neighbouring non-transfected cells. Quantifications are shown on the right panel; * *P* < 0.05, Student's t-test for paired data, n = 7.

The study of the molecular mechanisms underlying the potential effects of E2 on AMH expression were performed using the SMAT1 cell line, derived from a previously characterised immortalised cell line of mouse prepubertal Sertoli cells^23,25,33,72^. Since it had not been previously studied, we first showed that SMAT1 cells express neither ERα nor ERβ (Fig. 3A). Accordingly, incubation with E2 induced no changes in the activity of a 3,078-bp human *AMH* promoter (pGL2B-5′h*AMH*-3078) in luciferase assays (Fig. 3B).

By transfecting the expression plasmids pSG5-ERα or pSG5-ERβ, we achieved expression of both ERs (Fig. 3A). Next, we evaluated the responsiveness to E2 of SMAT1 cells transfected with pSG5-ERα or pSG5-ERβ, in terms of activation of pGL2B-5′h*AMH*-3078. Maximal activation of the *AMH* promoter was observed with E2 10^–10^ M in ERα-transfected and with E2 10^–7^ M in ERβ-transfected SMAT1 cells (Fig. 3C). In cells transfected with both ERs, the E2 response profile was similar to that observed in cells transfected with ERα alone. The subsequent experiments were performed using E2 10^–9^ M because that is the physiological intratesticular E2 concentration estimated in male adolescents and patients with CAIS^73^. The activity of pGL2B-5′h*AMH*-3078 was significantly increased in SMAT1 cells transfected with ERα, but not with ERβ (Fig. 3D). Therefore, we performed the rest of the experiments using ERα-transfected SMAT1 Sertoli cells.

We validated the effect of E2 on AMH protein expression in SMAT1 Sertoli cells transfected with pSG5-ERα and incubated with E2 10^–9^ M (Fig. 3E). The intensity of AMH immunofluorescent staining in individual cells, measured with ImageJ software, was significantly increased in ERα-expressing cells relative to neighbouring cells that did not express ERα. These results confirm that E2 10^–9^ M, through ERα, increases endogenous AMH expression in SMAT1 Sertoli cells.

### Effect of ERα agonists and antagonists on AMH promoter activity

**Figure 4:**
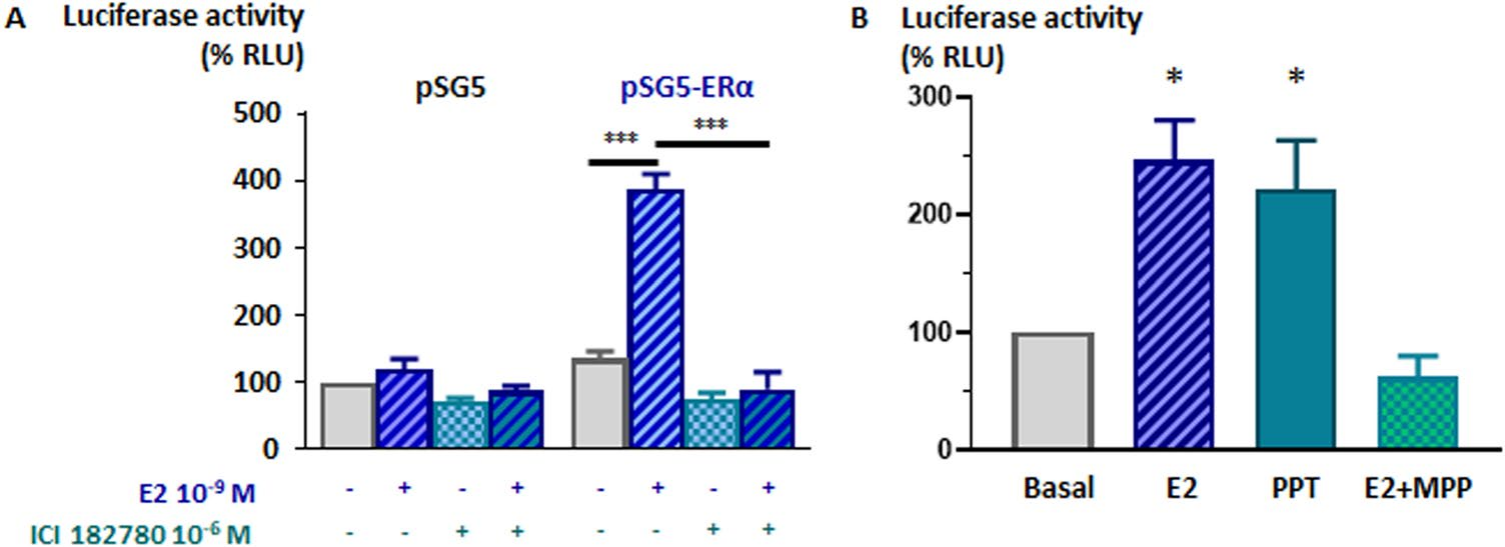
Effect of ER agonists and antagonists on hAMH promoter activity in SMAT1 cells. **(A)** Activity of pGL2B-5′hAMH-3078 in cells transfected with pSG5 or pSG5-ERα in basal conditions (considered as 100% of relative luciferase, RLU) and after incubation with E2 10^–9^ M and/or the selective antagonist ICI 182780 10^–6^ M. *** *P* < 0.001, analysis of variance (ANOVA), followed by Tukey's multiple comparison test, n = 8. **(B)** Activity of pGL2B-5′hAMH-3078 in ERα-transfected cells in basal conditions (considered as 100% of RLU) and after incubation with E2 10^–9^ M, the selective ERα agonist 10^–6^ M PPT or the selective ERα antagonist MPP 10^–8^ M. * *P* < 0.05, t-test for one sample compared to theoretical value of 100% (basal), n = 3.

We further verified that the effect of E2 10^–9^ M on the activity of the 3,078-bp h*AMH* promoter in SMAT1 Sertoli cells is mediated by ERα in luciferase activity assays after performing transient transfection experiments with pSG5-ERα and pGL2B-5′h*AMH*-3078-luc. The ERα and ERβ antagonist ICI 182780 significantly reversed the E2-induced h*AMH* promoter activity (Fig. 4A). PPT, a potent and selective ERα agonist, caused a significant stimulation of h*AMH* promoter activity while MPP, a silent and selective antagonist of ERα, induced a significant inhibition of the h*AMH* promoter activity (Fig. 4B). These results indicate that ERα mediates the increased h*AMH* promoter activity observed in SMAT1 Sertoli cells exposed to the E2 levels observed in human physiological/pathophysiological conditions.

### Human AMH promoter sequences responsive to E2

**Figure 5:**
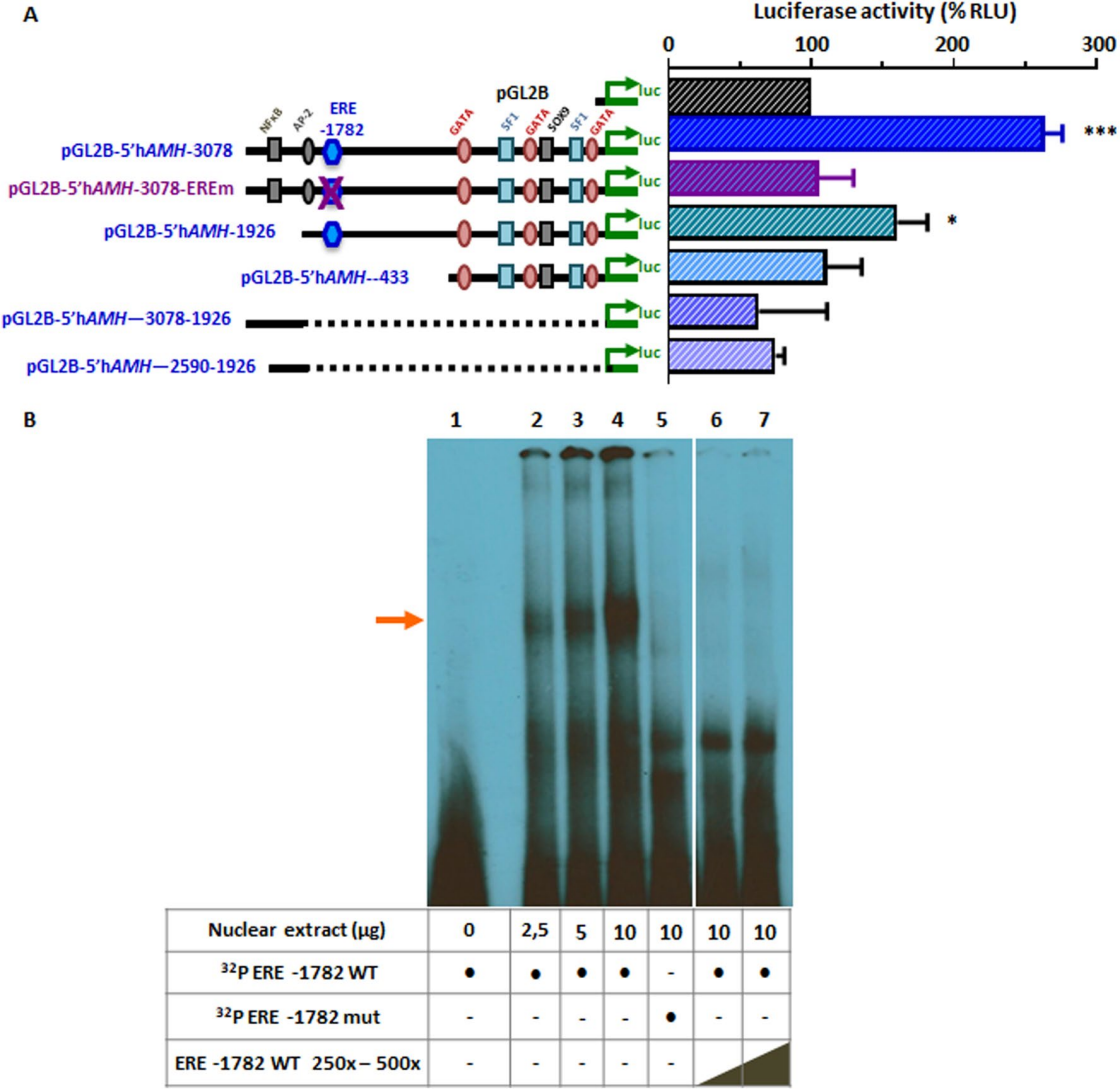
Relevance of the ERE site for E2 regulation of the h*AMH* promoter.** (A)** Luciferase activity of various pGL2B-5´hAMH constructs in SMAT1 Sertoli cells co-transfected with pSG5-ERα and incubated in basal conditions (considered as 100% of the relative luciferase units, RLU) or with E2 10^–9^ M. The percentage value reflects the response (E2/basal × 100). ERE: half-oestrogen response element present at position −1,782 of the human *AMH* promoter. * *P* < 0.05, *** *P* < 0.001, t-test for one sample compared to theoretical value of 100% (basal), n = 3. **(B)** Electro-mobility shift assay (EMSA) to test ERα binding to the hemi-ERE present at – 1,782 of h*AMH* promoter. Nuclear extracts from SMAT1 cells (between 0 and 10 µg) were incubated with a ^32^P-labeled DNA probe spanning the sequence of the wild-type (WT) or mutated (mut) hemi-ERE site and an excess (250 × or 500 ×) of ERE unlabelled WT probe. The arrow indicates the band corresponding to the WT ERE probe. Lane 1 does not contain nuclear extract. Lanes between 6 and 7, with irrelevant or duplicated experimental conditions, were cropped (for full-length blot, please see Supplementary material).

Next, we tested the involvement of the half-ERE site present at − 1,782 bp of the h*AMH* promoter in response to E2 in ERα-expressing SMAT1 Sertoli cells. The increased activity of the 3,078-bp h*AMH* promoter observed in response to E2 was abolished in SMAT1 cells transfected with a construct of the h*AMH* promoter carrying an inactivating ERE mutation (Fig. 5A). The response of the h*AMH* promoter to E2 was maintained, albeit at a somewhat lower level, when a 1926-bp promoter with an intact ERE was used. Conversely, no response was seen when *hAMH* promoters carrying exclusively the proximal 433 bp or sequences ranging from – 1,926 to − 3,078 bp or from – 1,926 to − 2,590 bp, i.e. lacking the ERE, were used.

The existence of a direct interaction between ERα and the half-ERE at – 1,782 of the h*AMH* promoter was tested in electro mobility shift assays (EMSA), performed with nuclear extracts from SMAT1 cells transfected with ERα and incubated with E2 10^−9^ M for 24 h prior to nuclear extraction (Fig. 5B). Complex formation observed with the ERE wild-type radioactive probes, but not the mutant ERE probes, was displaced with increasing concentrations of excess cold probe (250 × to 500 ×), which supports the specificity of the binding.

Taken together, these results indicate that E2 10^–9^ M, through ERα, increases the activity of the h*AMH* promoter in SMAT1 Sertoli cells, using the half-ERE site present at – 1,782 bp of the h*AMH* promoter. We cannot rule out that other sequences present upstream of – 1,926 are also involved in the activation of the h*AMH* promoter in response to E2.

### Involvement of GPER in hAMH promoter responsiveness to E2 in SMAT1 Sertoli cells

**Figure 6:**
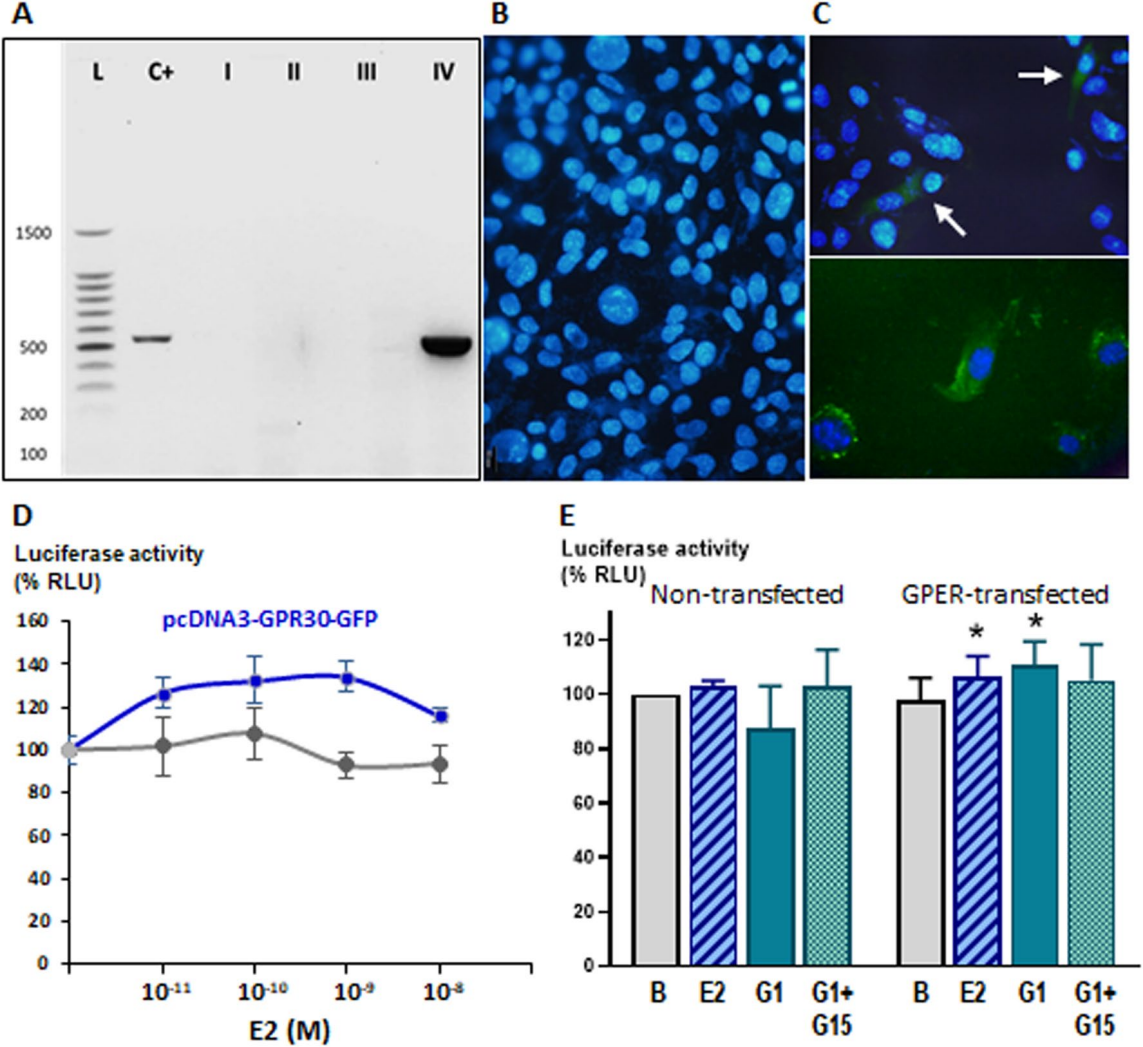
Relevance of GPER signalling in E2 regulation of the h*AMH* promoter. **(A–C)** Characterisation of GPER expression in SMAT1 cells: **(A)** electrophoresis after RT-PCR for *GPER*. L: 100-bp ladder, C + : positive control from human kidney cells, I: non- transfected SMAT1, II-IV: SMAT1 transfected with pcDNA3-GPR30-GFP (II: without RT enzyme, III: without cDNA): a ~ 540-bp band indicating *GPER* expression is observed in the transfected cells as well as in the positive control; **(B)** immunofluorescence in non-transfected SMAT1 cells; **(C)** immunofluorescence in SMAT1 transfected with pcDNA3-GPR30-GFP (arrows indicate positive cells, with higher magnification in the bottom figure). **(D)** SMAT1 cells transfected with pGL2B-5′hAMH-3078, and co-transfected with pcDNA3-GPR30-GFP were exposed to E2 from 0 to 10^–8^ M. The grey curve corresponds to SMAT1 cells not transfected with GPER. % RLU: relative luciferase units, considering the baseline condition (without GPER and without E2) as 100%. **(E)** Activity of pGL2B-5′hAMH-3078 in SMAT cells transfected or not with pcDNA3-GPR30-GFP in basal conditions (B, considered as 100% of relative luciferase, RLU) and after incubation with E2 10^–9^ M, the selective GPER agonist G-1 (100 nM) and the selective antagonist G-15 (100 nM). * P < 0.05, analysis of variance (ANOVA), followed by Sidak's multiple comparison test-all versus B (basal) for each set (non-transfected or GPER-transfected), n = 10.

GPER expression in Sertoli cells, described in rats^63^, is not conserved in SMAT1 cells (Fig. 6A,B). Therefore, to test whether GPER may mediate E2 action on h*AMH* promoter activity, we transfected SMAT1 cells with pcDNA3-GPR30-GFP^56^ expression vector for GPER (Fig. 6C) and incubated them with increasing concentrations of E2 (Fig. 6D). Maximal activity of the 3,078-bp h*AMH* promoter was seen with E2 10^–9^ M, corresponding to physiological oestrogen concentrations, as already mentioned.

The modest but significant effect of E2 on the activity of the 3,078-bp h*AMH* promoter mediated by GPER was also observed when E2 was replaced by G1, a potent and selective GPER agonist^63^, but reversed when the selective GPER antagonist G-15 was added (Fig. 6E). These results indicate that, in addition to ERα, GPER may also be involved in E2-induced increase of the h*AMH* promoter activity.

## Discussion

Testicular AMH production is increased in different clinical conditions characterised by elevated oestrogen levels. In this work, we show that E2 in the concentration range observed in those conditions is capable of inducing an upregulation of endogenous AMH protein expression as well as of the h*AMH* promoter activity in the prepubertal Sertoli cell line SMAT1. This process mainly involves ERα binding to a specific ERE sequence present at position – 1,782 of the h*AMH* promoter and also, more modestly, the membrane receptor GPER. At higher concentrations, the effect could also be attained via the ERβ receptor. The ERα and ERβ antagonist ICI 182780 downregulated h*AMH* promoter activity in SMAT1 cells and resulted in a decrease in serum AMH levels in prepubertal male mice. Finally, we confirmed that ERα expression is conserved in Sertoli cells in testicular tissue from patients with CAIS and both ERα and ERβ are expressed in Sertoli cells of normal prepubertal testicular tissue.

The main physiological role of AMH in mammals is the induction of Müllerian duct regression in the early stages of fetal life in males. Its persistent secretion by the testes after sex differentiation is completed and its production by the ovaries have led to the search for other biological functions for AMH. Regardless of the existence of other potential functions, AMH has become a useful clinical biomarker of testicular function in paediatric ages^74,75^, when circulating levels of testosterone and gonadotrophins—the typical markers of the male reproductive axis in adults—are low and may be of little clinical help^76^. The usefulness of AMH to interpret pathophysiological conditions has increased as the mechanisms underlying its production have been unveiled. The fact that basal AMH expression is independent of gonadotrophins explains that sex differentiation can occur before the fetal hypothalamic-pituitary–gonadal axis is functional, whereas its upregulation by FSH through Gsα protein signalling justifies the low AMH levels observed in newborns with congenital hypogonadotrophic hypogonadism^77,78^ and high levels in boys with McCune-Albright syndrome^22^. Downregulation of AMH expression by androgens, through AR interaction with the transactivating factor SF1^29^, underlies the decrease of serum AMH in both normal and precocious puberty in males^26^, whereas the lack of AR expression in the fetal and early infantile Sertoli cells^31–33^ explains why AMH remains high in spite of intratesticular androgen concentrations similar to those seen in adults and in boys with precocious puberty diagnosed before the age of 1 year^34^. In brief, the knowledge on the signalling pathways that regulate AMH testicular production have served to potentiate its use as a biomarker of gonadotrophin and steroid action in the prepubertal and pubertal testis^79^. In this work, we shed light on the molecular mechanisms that underlie AMH regulation by E2, providing insight into the potential biological processes that are responsible for the coincident increase in oestrogens and AMH in patients with hyperoestrogenic states, like CAIS^36,37^ and Peutz–Jeghers syndrome^43^.

Several observations led us to test the hypothesis that oestrogens induce an increase in AMH and not the opposite. On one hand, sequences compatible with a binding site for the ER have been described on the h*AMH* promoter^45^. On the other hand, in the ovaries AMH downregulates aromatase^80,81^, the enzyme responsible for E2 synthesis from testosterone, and E2 increases AMH expression^46^. Particularly in patients with CAIS who have not been gonadectomised before the age of puberty, FSH increases^82^ and acts on Sertoli cells which remain at an immature state owing to the lack of androgen action in spite of high testosterone production. FSH is known to activate aromatase in Sertoli cells^48,83^, which are then capable of metabolising testosterone to E2. In pubertal patients with CAIS, circulating E2 levels are between 20 and 250 pmol/l (i.e. in the 10^–10^ to 10^–11^ M range)^82^, and intratesticular concentrations are usually 400-fold higher (i.e. in the 10^–8^ to 10^–9^ M range)^73^. We exposed SMAT1 Sertoli cells to E2 at 10^–9^ M in our experiments in order to mimic CAIS conditions, thus supporting the biological plausibility of our hypothesis. According to our results, we privilege ERα and, to a lesser extent GPER, as the most likely mediators of oestrogen action on the *AMH* promoter activity, since ERβ only conveyed a significant response when SMAT1 cells were exposed to E2 at 10^–7^ M, i.e. a concentration tenfold higher than that expected within the testes. Our results indicate that there are differences between the Sertoli cell of the peripubertal testis and the granulosa cell of the adult ovary as regards to AMH regulation by E2. We show here that ERα seem to be the physiological mediator of E2 stimulatory effect on AMH expression whereas ERβ would be stimulatory only at supraphysiological levels. Conversely, in the adult ovary, ERα and ERβ show opposing effects, and the resulting *AMH* transcriptional activity depends on the ERα/ERβ expression ratio in granulosa cells^46^.

To support the physiological and pathophysiological relevance of our findings we verified the expression of ERs in testicular tissue from patients with CAIS. We could confirm that ERα, which we prioritised for the oestrogenic effect on AMH promoter activity, is present in Sertoli cells. Expression of ERα and ERβ in testicular tissue is controversial in the literature (for review, please see ref^58^). Our results are in keeping with those previously reported by Cavaco and colleagues^60^ using the same antibody, but intensity seems somewhat lower in the nuclei. Like for other steroid receptors, nuclear localisation may be weaker in prepubertal ages, when steroid levels are very low^32^. Other reports found no ERα staining in Sertoli and germ cells^59,84^. These divergent observations might be explained by the existence of different ERα isoforms^85^ and the capacity of the antibodies used to detect them by immunohistochemistry. GPER is also expressed in Sertoli cells, as previously shown in young males^61^. Unfortunately, the proven antibody used for immunohistochemistry in the referenced work was no longer available at the moment of our study.

One strength of our study is the use of SMAT1 cells as a model of prepubertal Sertoli cells. The expression of AMH declines significantly during pubertal development, and most Sertoli cell lines derived from adult testes do not express AMH^72,86^. SMAT1 cells maintain AMH expression, indicating that the basal transcriptional machinery for AMH is present^72^; indeed, its validity for the study of AMH regulation has been already proven^23–25,29,33,87^. However, SMAT1 cells have lost the expression of all oestrogen receptors, as shown in the present work. While this may seem a limitation, requiring transfection of oestrogen receptor expression vectors in all experiments, it represented an advantage to test specificity of our results since we could use exposure of non-transfected SMAT1 cells to E2, agonists and antagonists as a negative control. We verified the expression of oestrogen receptors in SMAT1 cells with various antibodies to ERα and ERβ. For GPER, we could not obtain reliable results with antibodies, hence we confirmed its expression by RT-PCR. The specificity of the oestrogen receptors types involved was probed by using different agonists and antagonists. ICI 182780 is a high affinity ERα and ERβ antagonist, devoid of any partial agonism on them^88,89^. PPT is a potent and selective ERα agonist^90^, while MPP is a silent and selective ERα antagonist^89^. Finally, G-1 is a potent and selective GPER agonist whereas G-15 is a selective antagonist, displaying no activity at ERα and ERβ^55^. The GPER-mediated effect was only moderate, up to 1.4-fold as seen in our dose-dependent experiments with E2 shown in Fig. 6D. This modest effect seems to be comparable with those previously shown for other markers of GPER-mediated oestrogen action in Sertoli cells, such as BCL2 and BAX^63^. Whether an additive effect in physiological conditions in Sertoli cells expression all three ERs exist could not be tested in our experimental conditions, requesting co-transfections of all receptors.

Using targeted mutagenesis to modify the ERE sequence present on the h*AMH* promoter^45^ and also previously validated promoter constructs of different sizes^23–25,29^, we could provide further insight into the molecular mechanisms that explain E2 regulation of AMH expression. In fact, only the h*AMH* promoter variants containing the ERE sequence at – 1,782 were responsive to E2 incubation in the presence of ERα. The short h*AMH* promoter of 433 bp and the constructs containing sequences ranging from – 1,926 to − 3,078 bp or – 1,926 to − 2,590 bp, i.e. lacking the ERE sequence at – 1,782 bp, as well as the 3,078-bp promoter carrying an inactivating mutation of the ERE sequence showed no response when SMAT1 cells were exposed to E2. These results were further supported by the EMSA experiments. Unfortunately, although we tested several experimental conditions, we could not show a displacement in EMSA when using anti-hERα antibodies, which could be explained by a poor antibody-protein complex ratio of human ERα in these experimental conditions.

To corroborate in vivo the effect of E2 on AMH production by Sertoli cells, we attempted various protocols of oestrogenisation in prepubertal male mice. Unfortunately, the attempts proved unsuccessful, as indicated by the serum levels of E2 found in the mice after sacrifice. The experimental design was based on the fact that testicular AMH production falls significantly after postnatal day 10 in male mice^19^; therefore, any treatment with E2 should be given very early after birth. Another important issue is that sufficient levels of steroids need to be attained within the testes for a response to be observed. Indeed, because steroids are produced within the gonads, their intratesticular concentration usually reaches levels that are 100–1,000-fold higher than those observed in circulation^73^, and the usual steroid doses used in treatments targeting peripheral tissues—like genitals, muscle and bone—are ineffective to induce changes in Sertoli cell AMH production^91^. This means that the dose of E2 needed to be injected was unusually high, and it proved impossible to attain in the small volume that could be injected to mice aged 4–8 days weighing less than 5 g. The maximal E2 dose we could use probably inhibited FSH in treated mice, which may explain a potential decrease in the endogenous E2 production by the gonads. Nonetheless, mice treated with the potent ERα- and ERβ-antagonist ICI 182780^88,89^, commercially known as fulvestrant (Faslodex^®^), showed a significant decrease in serum AMH levels, thus supporting our hypothesis that oestrogens are involved in testicular AMH output. Alternative explanations are possible since ICI 182780 has an agonist effect on GPER^92^, although it seems to be weaker than the antagonist effect on ERα and ERβ^93^. Although we focused on a direct transcriptional regulation of AMH by E2, other mechanisms may also be implicated, e.g. translocation of ERα to the cell membrane inducing the MAPK3/1 phosphorylation cascade^94^ and/or a Sertoli cell mass effect. In fact, ERα and ERβ^62,63,94^, as well as GPER^68^ have been shown to induce prepubertal Sertoli cell proliferation, which could result in higher serum AMH levels as observed in mice^23^ and humans^22^.

**Figure 7:**
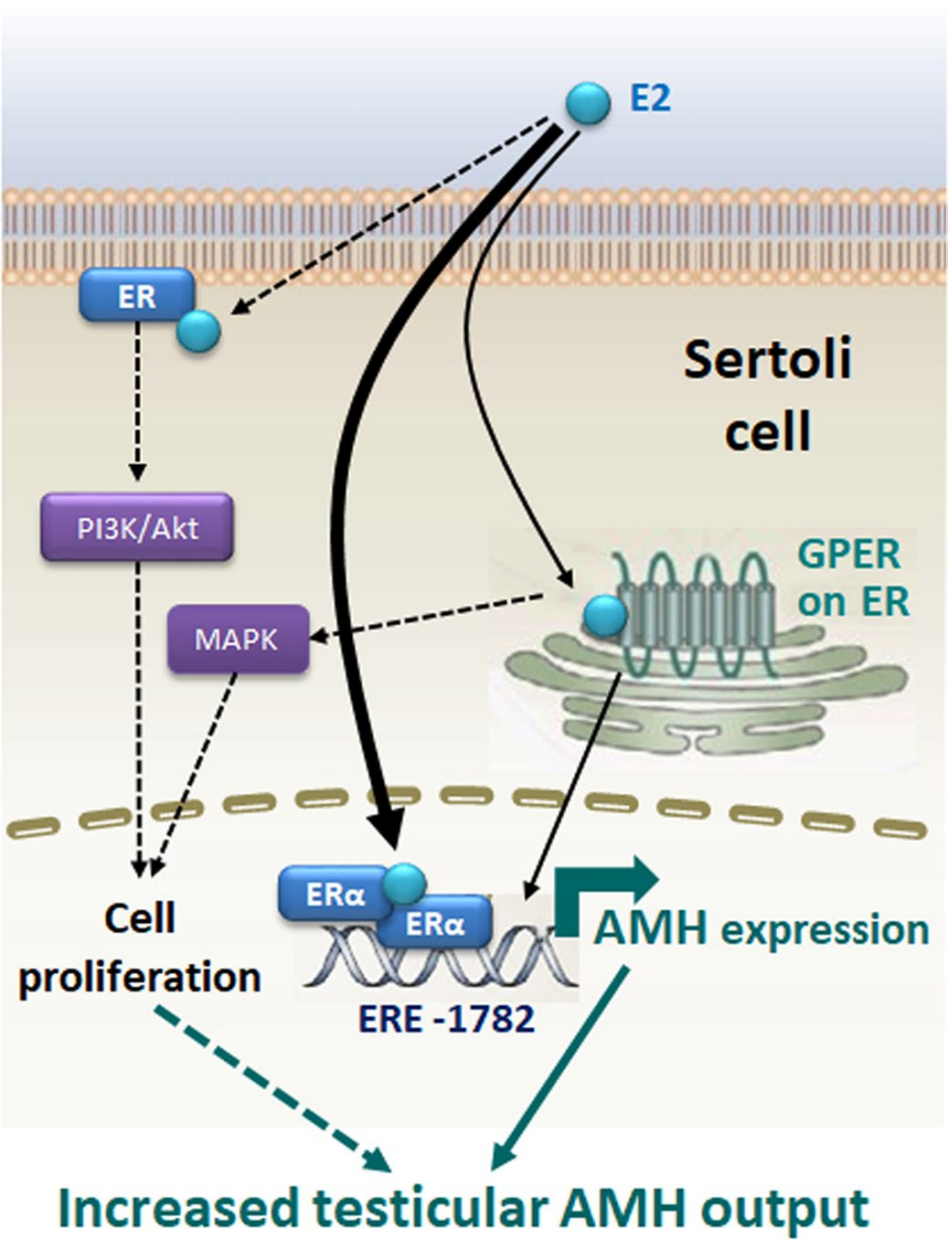
Proposed model for oestradiol (E2) regulation of the testicular AMH production in Sertoli cells. E2 upregulates AMH transcription mainly through nuclear oestrogen receptor α (ERα) binding to a specific oestrogen response element (ERE) on the *AMH* promoter, located 1,782 bp upstream of the translational start site. More modestly, GPER activation also upregulates *AMH* expression. The increased AMH expression results in a higher testicular AMH production. Another potential mechanism, not studied in this work, which could increase testicular AMH production is the increase in Sertoli cell proliferation induced by membrane-bound ERα, signalling through the PI3K/Akt pathway, and/or GPER, through MAPK signalling (dotted lines).

In summary, E2 upregulates AMH expression by increasing the activity of the h*AMH* promoter in the prepubertal Sertoli cell line SMAT1. Signalling through ERα, which binds to a specific ERE sequence present 1,782 bp upstream of the h*AMH* translation start site, seems to be the most relevant underlying mechanism. A more modest effect may also be conveyed through the membrane receptor GPER (Fig. 7). Other potential mechanisms include translocation of ERs to the plasma membrane with activation of MAPK3/1 and/or boosting of Sertoli cell proliferation. The evidence presented here provides biological plausibility to the proposal that testicular AMH production increases in various clinical conditions in response to elevated oestrogen levels.

## Methods

### Human tissues for immunohistochemistry

Testicular tissue from patients with the diagnosis of Complete Androgen Insensitivity Syndrome (CAIS) and from patients with acute lymphoblastic leukaemia was obtained from the archives of the Pathology Laboratory of CEDIE at Hospital de Niños Ricardo Gutiérrez. CAIS patients (n = 4) were 4 to 9 years-old, raised and females, and diagnosed due to either a familial history (sibling) with CAIS or as an unexpected finding during a surgery for hernia. As control of prepubertal testicular tissue, we used the normal testicular tissue present in biopsies routinely obtained before chemotherapy to rule out testicular compromise in boys with acute lymphoblastic leukaemia (n = 4), aged 4 to 7 years old. The study protocol was approved by the Institutional Review Board (Comité de Docencia e Investigación) and Ethics Committee (Comité de Ética en Investigación, protocol number CEI-17.01) of the Hospital de Niños Ricardo Gutiérrez de Buenos Aires. The study was performed in accordance with relevant international guidelines, such as the World Medical Association’s Declaration of Helsinki on Medical Research Involving Human Subjects, and Declaration of Taipei on Ethical Considerations Regarding Health Databases and Biobanks, and local regulations, such as law 3301/2009 of the Government of the City of Buenos Aires on Protection of Rights of Subjects in Health Research and its regulatory decree 58/2011, and the resolution 595/2014 of the Ministry of Health of the City of Buenos Aires on Requirements and Procedures Applicable to Behavioural, Socio-anthropological and Epidemiological Research Projects. Owing to its retrospective design with descriptive purposes and no anticipated effect on the prognosis or therapeutic management of the patients whose tissue samples were included, the need for a written informed consent was waived.

### Animals

Outbred CF-1 female pregnant mice (*Mus musculus*), obtained from Facultad de Ciencias Exactas y Naturales, Universidad de Buenos Aires, were kept in the animal facility of the Hospital de Niños Ricardo Gutiérrez on a 12:12 light–dark cycle, with food pellets and water ad libitum in cages, with their pups when born. Male offspring, used in the experiments, were sacrificed by cervical dislocation at postnatal day 9. Research using animals was performed in compliance with the Guide for the Care and Use of Laboratory Animals, 8th ed. Washington, DC: National Research Council, National Academies Press, 2011.

### Hormone assays

Serum was extracted and stored at − 20 °C until assayed. AMH was measured by enzyme-linked immunoassay using AMH Gen II ELISA^®^ (Beckman-Coulter Inc.), with a functional sensitivity of 2.5 pmol/l and intra- and inter-assay coefficients of variation of 9.4% and 10.5%, respectively. E2 was determined with an electrochemiluminescent assay (ECLIA, Roche Diagnostics GmbH), with a functional sensitivity of 10 pg/ml, an intra-assay coefficient of variation of 3.8% and an inter-assay coefficient of variation of 6.5%.

### Plasmids

The plasmids used in luciferase assays are shown in Supplementary Table S1.

### Site-directed mutagenesis

The plasmid of the h*AMH* promoter with the mutated ERE half-site was generated using the QuikChange II XL Targeted Mutagenesis Kit (Stratagene), as described^15^. Oligonucleotide primers for mutagenesis (Supplementary Table S2) were synthesised by Eurogentec (Liège, Belgium). Sequence changes were verified by direct sequencing, performed at Eurofins MWG (Ebersberg, Germany).

### Cell lines

SMAT1 cell line is a clonal line of immortalised Sertoli cells, obtained from pre-tumour testes of 6.5-day-old transgenic mouse expressing the SV40 oncogene under control of the *AMH* promoter^72^. This cell line has characteristics that make it extremely useful to study the physiology of the prepubertal Sertoli cell, maintaining the expression of both AMH and SOX9, GATA4 and SF1^23,25^. MCF-7 is a human breast cancer cell line that expresses high ERα levels^95^. KGN cell line was established from a human ovarian granulosa cell tumour highly expressing ERβ^96^. Human renal tubular epithelial cells (HRTEC) express GPER^97^.

### Reverse-transcriptase (RT) and polymerase chain reaction (PCR)

Expression of *GPER* was studied in SMAT1 cells by RT-PCR. Cells were grown in culture medium (as described above) in six-multi-well plates and RNA was obtained using TRI Reagent^®^ (Sigma T9424) following manufacturer’s instructions. Briefly, TRI Reagent^®^ was added to each well (1 ml per well) and incubated 5 min at room temperature. After collecting cell lysates, 200 μl of chloroform were added and incubated 2 min at room temperature. After centrifugation for 15 min at 15,000 × *g* at 4 °C, the aqueous phase was retrieved in clean tubes and 500 μl of isopropanol were added and incubated 10 min at room temperature and subsequently stored overnight at − 20 °C to increase RNA yield. After thawing at room temperature, samples were centrifuged for 60 min at 15,000 × *g* at 4 °C. Supernatants were discarded and pellets washed twice with ethanol 75% and once with ethanol 100%, followed by a 60 min centrifugation at 12,000 g at 4 °C. Ethanol was removed and pellets allowed to air-dry. The RNA pellets were resuspended with RNAse free water (20 μl final volume). Genomic DNA remnants were eliminated by treatment with RQ1 RNase-free DNase (Promega M6101). Concentration and quality of RNA was assessed by spectrophotometry. cDNA was obtained by reverse transcription using the MMLV I enzyme (Invitrogen) following manufacturer’s instructions. Briefly, 2 μg of RNA were used per reaction. Random hexamer primers (100 mM) and dNTPs (10 mM each, Invitrogen) together with the diluted RNA were incubated for 5 min at 65 °C and then placed on ice for at least 1 min. Subsequently, 7 μl of First Strand Buffer 5 × and DTT 0.1 M mix were added and incubated for 2 min at 37 °C. Finally, 1 μl of MMLVI (200 U) was added and incubated for 10 min at 25 °C followed by 50 min at 37 °C and 15 min at 70 °C. cDNA was stored at − 20 °C until use. RT-PCR was performed using an Eppendorf thermocycler.

Specific primers were designed for the detection of transcripts of the mouse *Gper1* and the human *GPER1* genes, using bioinformatics tools according to NCBI Blast https://blast.ncbi.nlm.nih.gov and Primer3plus program https://primer3plus.com. Primers were synthesised by Eurofins MWG|Operon (distributor in Argentina: Tecnolab). Primer sequences used are shown in Supplementary Table S3. A standard PCR was performed for amplification of the mouse and human cDNA, using 2 µg of cDNA per reaction and the GoTaq enzyme (Promega), following the manufacturer's instructions. Briefly, 5 µl of 5 × GoTaq Buffer with Mg^+2^, 0.5 µl of dNTPs (10 mM each), 1 µl of each primer (1 µM), 0.125 µl of GoTaq (5U/µl) and 3 µl of sample, in a final volume of 25 μl. A negative PCR control was included in which no sample was added and the volume was replaced by water. The cycling program consisted of an initial cycle of denaturation for 10 min at 94 °C, followed by 30 cycles of 30 s of denaturation at 94 °C, 30 s of alignment at 60 °C and 1 min of extension at 72 °C, followed by a final cycle of extension of 10 min at 72 °C.

### Cell culture and luciferase assays

SMAT1 cells were cultured at 37 °C in a humidified atmosphere with 5% CO_2_ in Dulbecco Modified Eagle Medium (Gibco 11995-065, Invitrogen) supplemented with MEM amino acids solution (Gibco 11130051), 10% fetal bovine serum (FBS, Gibco 16000-044), penicillin–streptomycin (final concentration 50 U/ml, Gibco 15070-063) and amphotericin B (final concentration 0.0025 mg/ml, Sigma A-4888).

For luciferase assays, SMAT1 cells were transiently transfected with 0.5 µg/well of luciferase reporter plasmids containing h*AMH* promoter length variants or mutants and 0.2 µg/well of ERα, ERβ or GPER expression plasmids, using Lipofectamine 3000 (Invitrogen) as suggested by the manufacturer. The plasmid pRL-TK (0.15 µg/well) was used as transfection control. Backbone plasmids were transfected to balance DNA amounts. Briefly, on day 1 SMAT1 cells were plated at 2 × 10^5^ cells/well in 24 multi-well plates. On day 2, medium was changed to medium without penicillin–streptomycin and amphotericin B for 2 h, and subsequently to 1% charcoal-stripped FBS-DMEM without phenol red, 1% MEM. The DNA-Lipofectamine solutions were added in a 1:2 DNA-Lipofectamine-Optimem (Gibco, 11058021) solution:transfection medium and incubated for 4 h. Transfection medium was then replaced by 10% charcoal-stripped FBS in DMEM, with amino acids, penicillin–streptomycin and amphotericin B, and incubated overnight. Cells were incubated with vehicle (ethanol), 17β-oestradiol (E2, Sigma E8875) or the following agonists or antagonists overnight in serum-free medium: (a) ICI 182780, a high affinity ERα and ERβ antagonist (IC50 = 0.29 nM), devoid of any partial agonism both in vitro and in vivo^88,89^, (b) PPT (4,4′,4′′-(4-Propyl-[1H]-pyrazol-1,3,5-triyl) trisphenol), a potent and selective ERα agonist with an effect 400-fold greater than on ERβ^90^, (c) MPP (1,3-bis (4-hydroxyphenyl)-4-methyl-5-[4-(2-piperidinylethoxy) phenol]-1H-pyrazoledihydrochloride), a silent, high-affinity selective ERα antagonist, with a 200-fold more powerful effect than on ERβ^89^, (d) G-1 (1-[(3aR*,4S*,9bS*)-4-(6-Bromo-1,3-benzodioxol-5-yl)-3a,4,5,9b-tetrahydro-3H-cyclopenta[c]quinolin-8-yl]-ethenone), a potent and selective GPER agonist, displaying no activity on ERα and ERβ at 10 µM (Tocris Bioscience)^55^, (e) G-15 [(3aS*,4R*,9bR*)-4-(6-Bromo-1,3-benzodioxol-5-yl)-3a,4,5,9b-3H-cyclopenta[c]quinoline], a potent and selective GPER antagonist, displaying no activity on ERα and ERβ at 10 µM (Tocris Bioscience)^55^.

Luciferase activity was measured following the recommendations of the supplier of the dual luciferase kit (Promega E1910), and renilla luciferase (pRL-TK) was used as a transfection control, as described^25^. After the culture medium was discarded and the cells were washed with PBS, 100 µl of PLB lysis buffer were added and incubated for 45 min at room temperature with shaking (120–150 rpm). Extracts were recovered, centrifuged at 14,000 rpm for 5 min at 4 °C. For luciferase activity assays, 45 µl of LAR II were added to 20 µl of supernatant from each sample and luminescence was measured on a Junior LB9509 luminometer (Berthold Technologies) or Synergy HTX multimode plate reader, coupled to software Gen5 (version 3.02, BioTek Instruments Inc.). For the measurement of renilla luciferase activity, 45 µl of Stop & Glo solution were added, and luminescence was measured. Results were expressed in relative luciferase units (RLUs), defined as the normalisation of firefly luciferase activity relative to renilla luciferase activity.

### Electro-mobility shift assays (EMSA)

EMSA was performed as previously described^15^. Briefly, 10^7^ SMAT1 cells plated in 75-cm^2^ flasks were transfected with 1 µg of pSG5-hERα and incubated with 10^−9^ M E2 for 24 h prior to nuclear extraction. For the preparation of the nuclear extracts, cells were rinsed twice with ice-cold PBS, resuspended in PBS and centrifuged at 1,000 × *g* for 5 min. The pellet was resuspended in buffer A containing protease inhibitors (10 mM HEPES pH 7.9, 1.5 mM MgCl_2_, 10 mM KCl, 0.5 mM dithiothreitol, 0.1% Nonidet P-40, Phenylmethylsulfonyl fluoride 1 mM, 2 mg/l leupeptin, 2 mg/l aprotinin). After centrifugation, the crude nuclear pellet was resuspended in buffer C (10 mM HEPES pH 7.9, 0.2 mM NaCl, 0.2 mM EDTA, 25% glycerol) and incubated for 20–30 min with shaking at 4° C. After separating the cell components by centrifugation at 12,000 × *g* for 5 min, the nuclear extracts were either directly evaluated or stored at – 80 °C. Protein concentration was determined with the Bradford Protein Quantification Assay (Bio-Rad).

For binding studies to the half-ERE site present at position – 1,782 of the h*AMH* promoter, double-stranded oligonucleotides (Supplementary Table S4) were end-labelled with γ-32P-ATP using T4 polynucleotide kinase (Promega). Unincorporated ATP was removed by column purification (Qiagen). Nuclear proteins (10 μg) were incubated with binding buffer (4% glycerol, 10 mM HEPES, pH 7.5, 1 mM dithiothreitol, 1 mM EDTA, 1 μg of poly dI-dC), 1 μg/μl BSA and the radiolabelled probe for 20 min at room temperature. Competition studies were performed in the presence of a molar excess of unlabelled probes. DNA–protein complexes were resolved by electrophoresis on a 5% non-denaturing polyacrylamide gel using Tris–borate-EDTA buffer. The gels were dried and subjected to autoradiography.

### Immunohistochemistry and fluorescence microscopy

For immunohistochemistry, 5–7-µm sections of testis tissues from gonadectomy were used. The samples were deparaffinised in xylene for 30 min and rehydrated by successive passages in ethanol of decreasing concentration (100%, 96% and 70% in distilled water). The activity of endogenous peroxidase was inhibited with 10% hydrogen peroxide in ethanol/70% distilled water for 20 min. After a series of washes with PBS and blocking with 10% fetal bovine serum in PBS for 1 h at room temperature in a humid chamber, sections were incubated with the primary antibodies overnight at 4 °C in a humid chamber. Primary antibodies are described in Supplementary Table S5. Incubation with a biotinylated secondary antibody (MultiLink^®^ HK268 1:50 concentrated biotinylated anti-immunoglobulins) diluted 1:50 in blocking solution (10 fetal bovine serum % in PBS) was performed for 1 h at room temperature in a humid chamber. Finally, after a series of PBS washes, the reaction was developed using Avidin Biotin Complex (ABC kit Vectastain, Vector) and 3–3′-diaminobenzidine (DAB, DAKO). The analyses were performed using a Nikon Eclipse E800 light microscope, and photos were taken with a Nikon digital machine associated with this microscope using the ACT-2U software.

The expression of endogenous AMH and the ERα in SMAT1 cells was evaluated by immunofluorescence. After incubation in the dark in a humid chamber for 1 h at room temperature with the corresponding secondary antibody (mouse or rabbit anti-immunoglobulin) coupled to Alexa Fluor^®^ 488 or Alexa-Fluor^®^ 594 (Abcam)^98^. Finally, the glasses were mounted with Vectashield (Vector) and sealed with enamel for observation with a Nikon Eclipse E800 microscope and image analysis with ImageJ software^99^.

The expression of GPER in SMAT1 cells transfected with the GPER expression vector pcDNA3-GPR30-GFP^56^ was verified by fluorescence microscopy in a Carl-Zeiss AxioScope A1 with filters for Hoechst 33342 (blue) and FITC (green). The images were obtained with the Axiocam ERc 5s camera and AxioVision LE software. Nuclei were counterstained with Hoechst 33342 (Invitrogen H1399).

### Western blots

Western blots were performed as previously described^100^. Briefly, SMAT1, MCF-7 or KGN cells or adult mouse uterine tissue were homogenised in TEGDS buffer with a polytron, after adding protease inhibitors. The homogenates were centrifuged for 10 min at 3,300 rpm at 4 °C, and the nuclei on the pellet were washed in TEDGS buffer plus 0.01% NP-40. The nuclei were resuspended in TEDGS containing 0.4 mol/l KCl and incubated at 4 °C for 30 min, and the nuclear homogenate was centrifuged at 12,000 rpm for 20 min at 4 °C. The nuclear extract was diluted 1:2 in TEDGS buffer with 30% glycerol, and proteins were quantified using the Lowry method. An amount of 100 μg total protein/lane was submitted to electrophoresis on 10% sodium dodecyl sulphate polyacrylamide gels (SDS-PAGE) using Laemmli's buffer system. After electrophoresis, blotting was performed onto a nitrocellulose membrane and blocked overnight in 5% dry skimmed milk dissolved in 0.1% PBS -Tween 20. After several washes, the membranes were incubated with the primary antibodies described in Supplementary Table S5 at room temperature for 2 h. Blots were probed with sheep anti-mouse or donkey anti-rabbit immunoglobulin, horseradish peroxidase-conjugated whole antibody (Abcam). The luminescent signal was generated with the ECL Western blotting detection reagent kit (GE Healthcare), and the blots were exposed to medical X-ray film for 10 s to 5 min.

### Statistical analyses

Relative Luciferase Units (RLU) were defined as the normalisation of firefly luciferase against renilla luciferase activity. Values are expressed as mean ± SEM for the percentage (%) RLU as compared to samples exposed to vehicle (basal condition). Student's t-test for unpaired samples, t-test for one sample compared to theoretical value of 100% (basal condition), or analysis of variance (ANOVA) followed by Tukey's multiple comparison test or Sidak's multiple comparison test were used as indicated in each figure legend. A difference was considered statistically significant when the *P*-value was < 0.05. All calculations were made using GraphPad Prism version 8 for Windows (GraphPad Software).

Sample size calculation was performed for the main outcome measures, as follow, based on pilot experiments. To show an increase of 50% in response to E2 through ERα or ERβ in transfected SMAT1 cells using a unilateral test, with a confidence level of 95% (α error < 0.05) and a statistical power of 80% (β error < 0.20), and an estimated variance of 900% for the unstimulated condition (control), a sample size of 4 was needed. For GPER, to show an increase of 15% in response to E2 using a unilateral test, with a confidence level of 95% and a statistical power of 80%, and an estimated variance of 144% for the unstimulated condition (control), a sample size of 8 was needed.

## Supplementary information

Supplementary Information.
