# Supplementary material for: Molecular mechanisms underlying AMH elevation in hyperoestrogenic states in males
Source: Sci Rep. 2020 Sep 15;10:15062. doi: 10.1038/s41598-020-71675-7 (PMC7492256; doi:10.1038/s41598-020-71675-7)

# Molecular mechanisms underlying AMH elevation in hyperoestrogenic states in males

Clara Valeri^1^, María M. Lovaisa^1^, Chrystèle Racine^2^, Nadia Y. Edelsztein^1^, Marina Riggio^3^, Sebastián Giulianelli^3,4^, Marcela Venara^1^, Patricia Bedecarrás^1^, María G. Ballerini^1^, Nathalie di Clemente^2^, Caroline A. Lamb^3^, Helena F. Schteingart^1^, Rodolfo A. Rey^1,5^

^1^Centro de Investigaciones Endocrinológicas “Dr. César Bergadá” (CEDIE), CONICET – FEI - División de Endocrinología, Hospital de Niños Ricardo Gutiérrez, C1425EFD Buenos Aires, Argentina.

^2^Sorbonne Université, INSERM, Centre de Recherche Saint Antoine (CRSA), IHU ICAN, 75012 Paris, France.

^3^Instituto de Biología y Medicina Experimental (IBYME-CONICET), C1428ADN Buenos Aires, Argentina.

^4^Instituto de Biología de Organismos Marinos, IBIOMAR-CCT (CENPAT-CONICET), U9120ACD Puerto Madryn, Argentina.

^5^Departamento de Biología Celular, Histología, Embriología y Genética, Facultad de Medicina, Universidad de Buenos Aires, C1121ABG Buenos Aires, Argentina.

Corresponding author: Rodolfo A. Rey ([rodolforey@cedie.org.ar](mailto:rodolforey@cedie.org.ar)). Centro de Investigaciones Endocrinológicas “Dr. César Bergadá” (CEDIE), CONICET – FEI - División de Endocrinología, Hospital de Niños Ricardo Gutiérrez, C1425EFD Buenos Aires, Argentina.

**SUPPLEMENTARY FILE**

## Supplementary Table S1. Plasmids used in this study.

| **Plasmid** | **Description** | **Reference** |
| --- | --- | --- |
| pGL2B | Empty vector carrying firefly luciferase gene | Promega |
| pGL2B-5´AMH-3078 | pGL2B with 3078 bp 5’ upstream of the TSS of the human *AMH* gene | ^23^ |
| pGL2B-5´AMH-2590 | pGL2B with 2590 bp 5’ upstream of the TSS of the human *AMH* gene | ^23^ |
| pGL2B-5´AMH-1926 | pGL2B with 1926 bp 5’ upstream of the TSS of the human *AMH* gene | ^23^ |
| pGL2B-5´AMH-433 | pGL2B with 433 bp 5’ upstream of the TSS of the human *AMH* gene | ^23^ |
| pGL2B-5´AMH-1926-2590 | pGL2B with sequences between -2590 and -1926 bp 5’ upstream of the TSS of the human *AMH* gene | ^23^ |
| pGL2B-5´AMH-1926-3078 | pGL2B with sequences between -3078 and -1926 bp 5’ upstream of the TSS of the human *AMH* gene | ^23^ |
| pGL2B-5´AMH-3078 EREm | pGL2B with 3078 bp 5’ upstream of the TSS of the human *AMH* gene with ERE site mutated at -1782 |  |
| pSG5 | Expression vector (empty) | Agilent |
| pSG5-hERα | pSG5 with cDNA of human ERα, kindly provided by Dr. J.A. Gustafsson, Stockholm | ^89^ |
| pSG5-hERβ | pSG5 with cDNA of human ERβ, kindly provided by Dr. J.A. Gustafsson, Stockholm | ^89^ |
| pcDNA3 | Expression vector (empty) | Invitrogen |
| pcDNA3-GPR30-GFP | pcDNA3 with cDNA of human *GPER* gene (GPR30), kindly provided by Dr. E.R. Prossnitz, Albuquerke | ^65^ |
| pRL-TK | Renilla luciferase expression vector. Transfection control. | Promega |

**Note:** For *AMH* promoter constructs site notation according to Picard *et al.*^90^ was used, reflecting a 10-bp to the 5’ displacement when compared to previous publications^23-25^.

**Abbreviations:** TSS, transcription start site.

## Supplementary Table S2. Oligonucleotide sequences used for site-directed mutagenesis of the ERE site present in the human AMH promoter.

| **Oligonucleotide** | **Sequence** | **Reference** |
| --- | --- | --- |
| ERE -1782 WT | S: GATGGTCGCCCTGAGGTCACAGGGACGAGGAGCCCTCTCTGTC  AS: GACAGAGAGGGCTCCTGCTCCCTGTGACCTCAGGGCGACCATC | ^44^ |
| ERE -1782 mut | S: GATGGTCGCCCTGAGGGCGGTACCACGAGGAGCCCTCTCTGTC  AS: GACAGAGAGGGCTCCTCGTGGTACCGCCCTCAGGGCGACCATC | -- |

**Note:** For *AMH* promoter constructs site notation according to Picard *et al.*^90^ was used, reflecting a 10-bp to the 5’ displacement when compared to previous publications^23-25^.

**Abbreviations:** S: sense; AS: anti-sense; WT: wild type; mut: mutation.

## Supplementary Table S3. Oligonucleotide primer sequences used for RT-PCR of mouse and human GPER.

| **Oligonucleotide** | **Sequence** | **Product** |
| --- | --- | --- |
| *Gper* (mouse) | S: CCCCAAAGTGCTGCAAGTCC  AS: TTTTCTGTTGGGTGTCCTGATCTGT | 108 bp |
| *GPER* (human) | S: ATGACCATCCCCGACCTGTA  AS: GACGAAGAAGACCAGCACCA | 540 bp |

**Abbreviations:** S: sense; AS: anti-sense.

## Supplementary Table S4. Oligonucleotide sequences used for EMSA studies of ERE site present in the human AMH promoter.

| **Site** | **Sequence** | **Reference** |
| --- | --- | --- |
| ERE -1782 WT | S: CGCCCTGAGGTCACAGGGACGAGGAGCCCT  AS: AGGGCTCCTGCTCCCTGTGACCTCAGGGCG | ^44^ |
| ERE -1782 mut | S: CGCCCTGAGGGCGGTACCACGAGGAGCCCT  AS: AGGGCTCCTCGTGGTACCGCCCTCAGGGCG | -- |

**Note:** For *AMH* promoter constructs site notation according to Picard *et al.*^90^ was used, reflecting a 10-bp to the 5’ displacement when compared to previous publications^23-25^.

**Abbreviations:** S: sense; AS: anti-sense; WT: wild type; mut: mutation.

## Supplementary Table S5. Antibodies used for immunohistochemistry or Western blots.

| **Antibody** | **Description** | **Supplier** | **Reference** |
| --- | --- | --- | --- |
| HC-20  (1/50) | Rabbit polyclonal antibody against an epitope located at the C-terminus of human ERα | Santa Cruz | sc-543 |
| MC-20  (1/50) | Rabbit polyclonal antibody against an epitope located at the C-terminus of mouse ERα | Santa Cruz | sc-542 |
| F-10  (1/50) | Mouse monoclonal antibody against an epitope located at the C-terminus of human ERα | Santa Cruz | sc-8002 |
| SP1  (1/50) | Rabbit monoclonal antibody against an epitope located at the C-terminus of human ERα | Invitrogen | MA5-14501 |
| 1531  (1/100) | Mouse monoclonal antibody against amino acids 256-505 of human ERβ | Santa Cruz | sc-53494 |
| Y-19  (1/100) | Goat polyclonal antibody against an epitope located at the N-terminus of mouse ERβ | Santa Cruz | sc-6821 |
| L42-43  (1/1000) | Rabbit monoclonal antibody against human recombinant AMH | In-house | ^19^ |

In parentheses, the dilutions used for Western blots, immunohistochemistry or immunofluorescence.

***Figure S1.*** Full length of blot in Figure 5B

Uncropped original image. Membrane was cropped to eliminate uninformative or irrelevant lanes. Specific areas of the original blot cropped for the figure are shown using dotted lines.


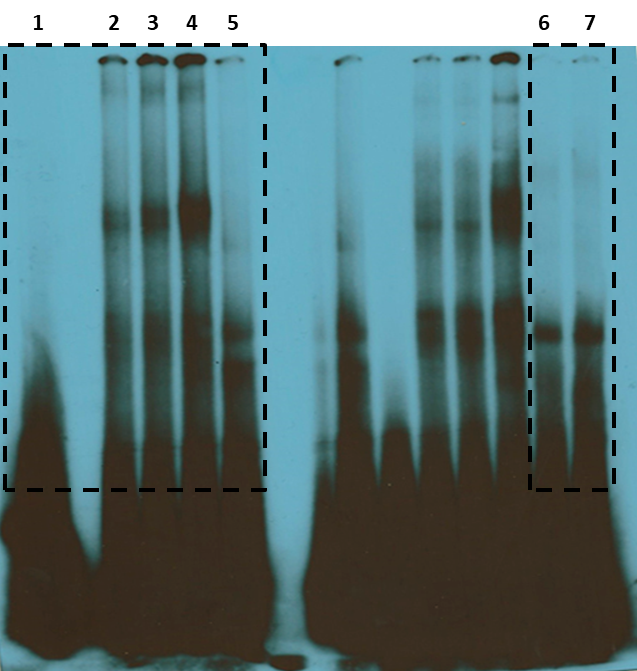

Supplement: Supplementary file 1 — Supplementary Information. [file 41598_2020_71675_MOESM1_ESM.docx]
